# Determinants of maternal healthcare utilisation among pregnant women in Southern Ethiopia: a multi-level analysis

**DOI:** 10.1186/s12884-023-05414-x

**Published:** 2023-02-04

**Authors:** Mekdes Kondale Gurara, Veerle Draulans, Jean-Pierre Van Geertruyden, Yves Jacquemyn

**Affiliations:** 1 Department of Public Health, College of Medicine and Health Sciences, Arba Minch University, Arba Minch, Ethiopia; 2 Faculty of Medicine and Health Sciences, Global Health Institute, University of Antwerp, Wilrijk, Belgium; 3 Faculty of Social Sciences, Centre for Sociological Research, KU Leuven, Leuven, Belgium; 4 Department of Obstetrics and Gynaecology, Antwerp University Hospital, UZA, Antwerp, Belgium

**Keywords:** Antenatal care, Delivery service, Ethiopia, Maternal health, Multilevel, Postnatal care

## Abstract

**Background:**

Despite efforts to make maternal health care services available in rural Ethiopia, utilisation status remains low. Therefore, this study aimed to assess maternal health care services’ status and determinants in rural Ethiopia.

**Methods:**

The study used quasi-experimental pre- and post-comparison baseline data. A pretested, semi-structured, interviewer-administered questionnaire was used to collect data. A multilevel, mixed-effects logistic regression was used to identify individual and communal level factors associated with utilisation of antenatal care (ANC), skilled birth attendance (SBA), and postnatal care (PNC). The adjusted odds ratio (AOR) and corresponding 95% confidence intervals (CI) were estimated with a *p*-value of less than 0.05, indicating statistical significance.

**Results:**

Seven hundred and twenty-seven pregnant women participated, with a response rate of 99.3%. Four hundred and sixty-one (63.4%) of the women visited ANC services, while 46.5% (CI: 42–50%) of births were attended by SBA, and 33.4% (CI: 30–36%) had received PNC. Women who reported that their pregnancy was planned (aOR = 3.9; 95% CI: 1.8–8.3) and were aware of pregnancy danger signs (aOR = 6.8; 95% CI: 3.8–12) had a higher likelihood of attending ANC services. Among the cluster-level factors, women who lived in lowlands (aOR = 4.1; 95% CI: 1.1–14) and had easy access to transportation (aOR = 1.9; 95% CI: 1.1–3.7) had higher odds of visiting ANC services. Moreover, women who were employed (aOR = 3.1; 95% CI: 1.3–7.3) and attended ANC (aOR = 3.3; 95% CI: 1.8–5.9) were more likely to have SBA at delivery. The likelihood of being attended by SBA during delivery was positively correlated with shorter travel distances (aOR = 2.9; 95% CI: 1.4–5.8) and ease of access to transportation (aOR = 10; 95% CI: 3.6–29) to the closest healthcare facilities. Being a midland resident (aOR = 4.7; 95% CI: 1.7–13) and having SBA during delivery (aOR = 2.1; 95% CI: 1.2–3.50) increased the likelihood of attending PNC service.

**Conclusions:**

Overall, maternal health service utilisation is low in the study area compared with the recommended standards. Women’s educational status, awareness of danger signs, and pregnancy planning from individual-level factors and being a lowland resident, short travel distance to health facilities from the cluster-level factors play a crucial role in utilising maternal health care services. Working on women’s empowerment, promotion of contraceptive methods to avoid unintended pregnancy, and improving access to health care services, particularly in highland areas, are recommended to improve maternal health service utilisation.

**Supplementary Information:**

The online version contains supplementary material available at 10.1186/s12884-023-05414-x.

## Background

Maternal mortality remains a global health challenge and is amongst the health indicators with the highest disparities between high-income countries and low-and middle-income countries (LMICs). The World Health Organisation (WHO) reported that approximately 810 women died daily in 2017 from preventable causes related to pregnancy and childbirth [1]. Although maternal mortality reduction remained a top priority on the global agenda among the United Nations’ Millennium Development Goals (MDGs) in 2000 and Sustainable Development Goals (SDGs) in 2015, LMICs have not made significant progress towards these goals. Overall, disparities between high-income and LMICs remain, as 94% of the deaths occur in LMICs, two-thirds of which were in Sub-Saharan Africa (SSA) alone [1, 2]. As maternal and neonatal health are linked inextricably, neonatal mortality, like maternal mortality, is very high in LMICs. Every day, an estimated 6,700 new-borns die worldwide, with SSA experiencing the highest rate of neonatal deaths [3].

Evidence indicates that three-fourths of maternal deaths in developing countries, including Ethiopia, were caused by five direct obstetric causes: haemorrhage; sepsis; unsafe abortions; obstructed labour; and hypertensive disorders from pregnancy. These direct causes of maternal deaths largely are preventable and could be avoided if all women had access to timely MHC (Maternal Health care) services [4, 5]. The WHO recommends that all pregnant women receive a minimum of eight ANC contacts, with the first occurring within the first three months of pregnancy, professional assistance during childbirth, and at least three PNC visits for better pregnancy outcomes [5, 6]. Given that most maternal and neonatal deaths occur during or shortly after delivery, professional assistance during childbirth is a crucial intervention for preventing stillbirths and improving newborns’ survival rates. Unfortunately, many women in developing countries do not have access to and use these recommended services [5, 7].

Even though promising improvements have been made in maternal and neonatal health in Ethiopia between 2000 and 2019, MHC service utilisation remains low compared with other SSA countries [8]. According to the Ethiopian Demographic and Health Survey (EDHS) report, professional assistance during childbirth increased from 5% in 2000 to 48% in 2019, and maternal mortality dropped by 50% between 1990 and 2015 [9, 10]. However, according to UN estimates, Ethiopia's MMR was estimated to be 401 deaths per 100,000 live births in 2017. In absolute numbers, Ethiopia has reported about 14,000 maternal deaths yearly, placing the country among the three countries (including the Democratic Republic of Congo and the United Republic of Tanzania) with over 10,000 maternal deaths in 2017. Furthermore, according to the 2019 Ethiopian DHS report, although 74% of pregnant women had at least one ANC visit with a skilled provider, only 43% had the recommended four visits, and 48% had given birth to their most recent babies in health facilities [9].

Previous studies on MHC service utilisation revealed like behavioural, socioeconomic, and healthcare-related factors associated with MHC utilisation. In addition, MHC service utilisation in Ethiopia varies depending on the mother’s awareness of danger signs during pregnancy, childbirth, and postpartum and her participation in decision-making [11]. Moreover, according to studies conducted in rural parts of Ethiopia, the long travel distance across steep terrain and poor road conditions considerably reduces the likelihood of giving birth at healthcare facilities [12]. Furthermore, in several studies, birth preparedness and complication readiness (BPCR) practice has been demonstrated to be among the strongest determinants of using institutional delivery services [11, 13, 14].

However, very little research is available in the context of rural Gamo Zone, Southern Ethiopia. Moreover, the few existing studies that focused entirely on ANC, urban women, or specific geographical areas [14–19] have not considered hierarchies by allowing for residual components at different levels in the hierarchy [11, 12, 20]. Therefore, a need exists for a clear understanding of contextual factors while employing appropriate methodology for a more comprehensive and accurate analysis to tailor contextual interventions [21]. As a result, all potential low-MHC service utilisation sources can be identified to design maternal service strategies at different levels. Therefore, the objective of this study was to assess the individual and community-level factors influencing the utilisation of MHC services. The study findings will help policymakers implement interventions to increase MCH service utilisation and promote maternal health in rural Ethiopia.

## Methods

### Study design, area and population

**Fig. 1:**
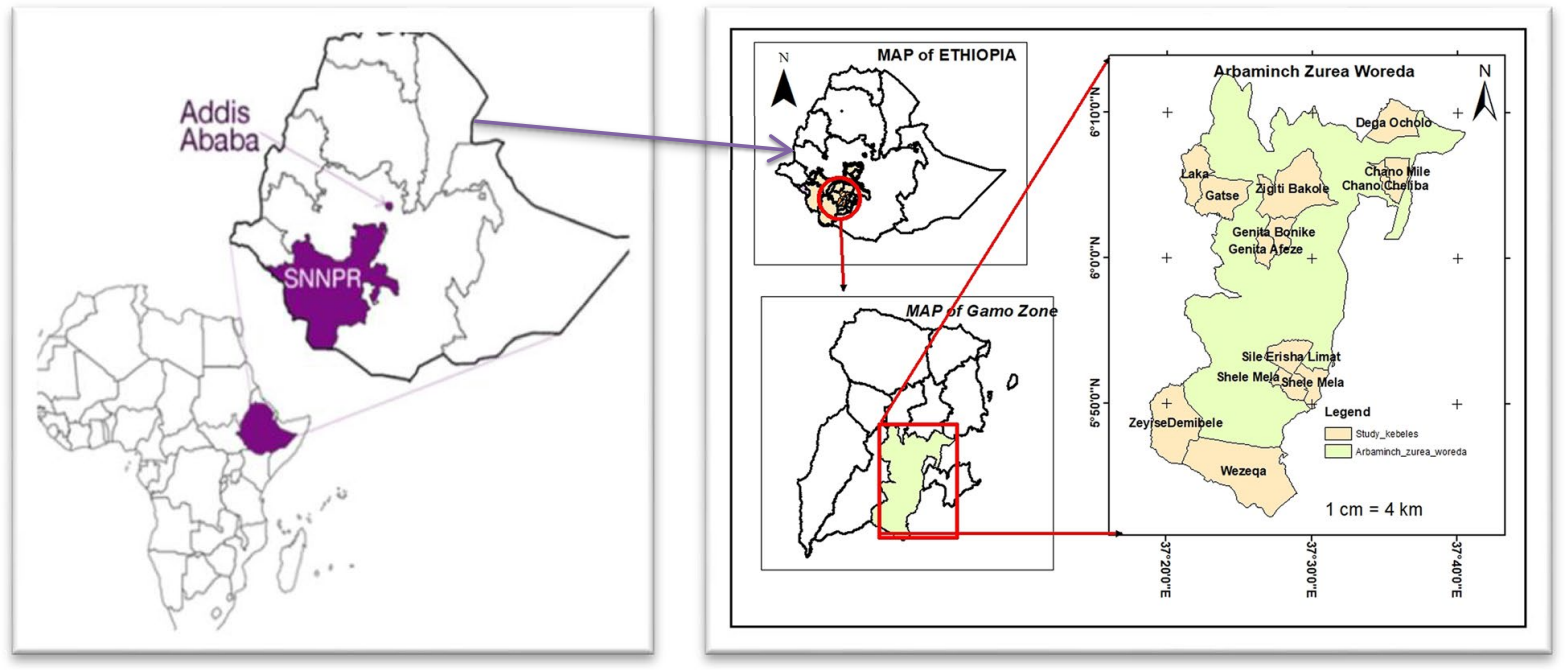
Map of the study area

We analysed data from a baseline survey from a quasi-experimental pre- and post-comparison study. Ten villages (*kebele*) were selected randomly from 29 villages of Arba Minch zuria district after stratifying the district in climatic zones in to high land and low land areas. Arba Minch town, Gamo Zone’s capital, is located 502 kms south of Addis Ababa, Ethiopia’s capital (Fig. 1). The total population of the district for 2017 was 195,858, with 50% of the population being female. Nine of the 10 villages included in the study were from the Arba Minch Health Demographic Surveillance site (AM-HDSS). This population primarily engages in subsistence agriculture, crop farming and small-scale animal rearing.

Similar to the rest of Ethiopia, Gamo Zone uses a three-tier health service delivery system, comprising primary, secondary and tertiary levels of care. The primary health care unit comprises a health centre with up to five health posts attached to it. The district has seven health centres and 40 health posts. At the health centre level, basic emergency obstetric care services are offered. Typically, health officers, midwives and nurses staff each health centre, and when they are faced with obstetric complications, they refer cases to Arba Minch General Hospital, where comprehensive emergency obstetric care is offered. The target population comprises pregnant women from Arba Minch Zuria district in Gamo Zone who had at least one birth in the past five years preceding the survey.

### Study variables

Three maternal health care service utilisation were considered – having received ANC during the pregnancy of the most recent birth, having given birth to the previous baby at a health facility and receiving any postnatal care following the previous childbirth – which were viewed as primary outcomes. We assessed predictors of each outcome separately, with respect to the previous birth. Explanatory factors were found at three levels: individual; household; and community. The use of ANC, BPCR awareness and BPCR practices were included as explanatory factors while examining factors linked with SBA. Similarly, utilisation of ANC and SBA, along with the other variables, was included in the analysis of PNC utilisation. Each explanatory variable’s coding is provided in Table 1.

**Table 1:** Description of the variables and measurements for the multilevel logistic regression analysis of the determinants of maternal health care utilization in southern Ethiopia

Variables	Descriptions	Measurements
**Dependent variable**		
ANC	Proportion of women who had received antenatal care	A woman who had attended antenatal care was coded as “1” and who did not use as “0”
Delivery (SBA)	Proportion of women who gave birth in a health facility	A woman who had given birth in a health facility was coded as “1” and who did not use as “0”
Post natal care	Proportion of women who received any postnatal care after delivery	A woman who had received any postnatal care after delivery was coded as “1” and who did not use as “0”
**Level 2 (higher level) independent variables**		**Communal (cluster level) variables**
Climatic zone	The usual place of residence where the woman lives	The three common agro ecological zones Highland, Midland and Lowland
Travel time to the nearest BEmONC center	Travel time estimated by the data collectors to the nearest health center	A woman who had to travel less than 2 h was coded as “1” and who travelled greater distance as “0”
Easiness to arrange transportation in case of emergency	Perception on easiness to arrange transportation in case of emergency	A woman who reported easiness to arrange transportation was coded as “1” and difficulties as “0”
**Level-1 (lower-level) independent variables**		**Individual level variables**
Woman’s age	Age of women at interview in completed years	Collected in absolute number and latter categorized as a binary variable 15–29 years old and 30–49 years old
Wealth quintiles	House hold assets, home, land, and livestock’s ownership were assessed, and wealth index was computed by using principal component analysis	The wealth status was categorized into five groups, then ranked from the poorest to the wealthiest quintile
Woman’s education	Highest level of education attained by the respondent	Ordinal variable based on the highest level of education attained: No formal education, primary education, and secondary or higher
Mother’s occupation	The proportion of women who worked in addition to household chores	Mother’s occupation was categorized into 2 groups: unemployed, farmers/agriculture workers/laborers
Religion	The religious background of the respondent	Each religion was entered and later recoded as “Orthodox Christian”” “Protestant” and “Others.” Others were merged because they were very few for logistic regressions
Gravidity	Number times the women got pregnant ever	Collected in absolute number and latter categorized into “1–2 pregnancy,” and “ ≥ 3 pregnancy”
Pregnancy wontedness	Whether pregnancy was wanted or mistimed	Binary variable distinguishing women who reported that their pregnancy was desired at the time (coded as 1)and those who stated that they either did not want to have any more children or “would have loved to wait” for some time before conceiving (coded as 0)
Attitude towards Skilled care	On a three-point Likert scale, ten (10) questions were completed to assess respondents' attitudes (Cronbach's alpha value of 0.94, internally consistent and reliable	Mean score was computed for the composite attitude score; those scored above or equal to the mean score were categorized into having “Good attitude,” and those who scored less than the mean were categorized a having “Poor attitude”
Awareness on danger signs	The proportion of women who knew the danger signs during pregnancy, childbirth and postpartum period	those who mentioned three of the danger signs were categorized as having “good awareness” and those who mentioned less than two were categorized as “having poor awareness”
Awareness on BPCR	The proportion of women who knew the BPCR activities	Those who mentioned three of the BPCR activities were categorized as having “good awareness” and those who mentioned less than two were categorized as having poor awareness
Practice BPCR	The proportion of women who prepared for birth and its complication	The participants who fulfilled at least three of the basic criteria on the BPCR index were considered as “well prepared” and the rest of them were “less prepared”

### Sample size

The sample size was estimated based on quasi-experimental study design. SBA’s prevalence in an Ethiopian rural setting was estimated to be 43% from EDHS 2019 [9]. The sample was based on 80% power to detect a change of 10% (with a 5% error level) and a design effect of 1.5. A sample size of 50 pregnant women per cluster was required, and accordingly, the final calculated sample size after considering a 10% loss to follow-up was 392 subjects per group (784).

The study included all nine villages of the Arba Minch-HDSS and one village from the Arba Minch Zuria district. Table 2 provides the characteristics of the 10 clusters/*kebele* included in the study. AM-HDSS was modelled after a stratified (agro-ecology), two-stage, cluster-sampling technique. We conducted a census to identify all eligible pregnant women from the selected villages and identified 1,447 women who were pregnant in 2017 from 10 villages (< 27 weeks of gestation). Altogether, 732 who fulfilled the inclusion criteria were interviewed for the study. Individuals who did not have a plan to move from where they were living until the end of the follow-up period were included in the baseline study (727), and they were all married.

**Table 2:** The character of the eleven clusters /kebele included in the study, Gamo zone 2020

S.N	Cluster	Total population	Number of HHs*	Number of health facilities	Main sources of income	Transport access to health center	Community media exposure	Agro ecology zone
1.	Zigity Bakole	7372	1474	2	Farming	Easy	Low	Highland
2.	Gatse	11,994	2390	2	Farming	Easy	Low	Highland
3.	Laka	9037	1847	1	Farming	Difficult	Low	Highland
4.	Zigity Pereso	3533	706	1	Farming	Difficult	Low	Highland
5.	Genta Bonke	6243	1274	2	Farming	Difficult	Low	Highland
6.	Zeyse Dembile	1605	328	2	Farming	Easy	Low	Midland
7.	Wezeqa	5992	1223	1	Farming	Easy	Medium	Midland
8.	Dega Ocholo	4175	835	1	Farming	Difficult	Low	Highland
9.	Dega Chenge	7431	887	1	Farming	Difficult	Low	Highland
10.	Shelle Mella	7313	1493	1	Mixed	Easy	Medium	Lowland
11.	Chano Chalba	7699	1812	1	Mixed	Easy	Medium	Lowland

^*^Number of Households per cluster

### Data collection tool, procedure and personnel

The data were collected using a pretested, semi-structured, interviewer-administered questionnaire comprising five main components: socioeconomic and demographic variables; obstetric characteristics; birth preparedness and complication readiness (BPCR) practices; maternal health care characteristics (places of births, number of ANC and PNC visits); and attitudes towards the importance of skilled birth attendance and maternity waiting homes. The EDHS suggested that household-asset questions be used to construct the household wealth index [9]. Face-to-face interviews were conducted in the local language by trained data collectors who received three days of training. Eleven data collectors and six site supervisors working in the district participated in the data collection. Data were collected on a smartphone-based application, Open Data Kit (ODK). After a completeness check, the data were uploaded to a secure database in ODK Aggregate, and ODK briefcase software was used to retrieve the data in Comma Separated Variables file format. Data were cleaned and analysed using Stata (StataCorp, Version 16, College Station, TX, USA). The questionnaire was written in English, then translated into Amharic and back to English to ensure that the translation accurately represented the original meaning. Before data collection, the questionnaire was pre-tested in a village in the Arba Minch Zuria district, whose demographic characteristics resembled those of the sample population, then modified based on the results. Inter-item consistencies between the variables were tested for the variables creating each component of items to measure participants attitude towards skilled birth attendance using the Cronbach’s alpha (all variables were > 0.86).

### Data management and analysis

Sociodemographic, economic, and obstetric data were presented using descriptive statistics. Bivariate analysis was also used to look into the relationship between the outcomes and the explanatory variables. For the multi-level analysis, we included variables with a p-value of 0.05 in the bivariate analyses with 95% CI. A separate multi-level model was constructed for every one of the three outcome variables. Due to the clustering of the data—individuals were nested within households, and households were nested in communities—we have chosen a multi-level analysis instead of conventional logistic regression.

### Model building and parameter estimations

Given that no two communities, or kebeles, have the same variance, the likelihood of MHC uptake differs significantly among communities. Therefore, a four-level multilevel model was fitted based on these attributes of the data. The first level was the null model (Model 0), which had no exposure variables and was designed to look for community variance and provide evidence for assessing the random effects of MHC utilisation at the community level. Model I was a multivariable model that was adjusted for characteristics at the community level; Model II incorporated factors at the individual level; and Model III, the final model, was fitted by taking this datum characteristic into account both individual and community-level factors as the outcome variable. A forward stepwise approach was followed until we reached the final model.

The measure of association (fixed effects) was estimated and expressed as an aOR with a 95% CI. Regarding the measures of variation (random effects), community-level variance, intra-cluster correlation coefficient (ICC), and median odds ratio (MOR) were used. The ICC, which quantifies the proportion of observed variation (variance partitioning) in the outcome that is attributable to the effect of clustering and was computed using the formula ICC = Va/(Va + π^2^/3), where: Va community level variance and the unobserved individual variable follows a logistic distribution with individual level variance equal to π^2^/3 (i.e., 3.29) [22].

We have also calculated the median odds ratio (*MOR* = *exp( √*^*2*^ × *Φ*^*–1*^* (*0.6745*)))* [23] to quantify the variation between clusters by comparing two persons with the same covariates from two randomly chosen, different clusters. Where *Φ(·)* is the cumulative distribution function of the normal distribution with mean 0 and variance 1, Φ^–1^(0.75) is the *75*^*th*^* percentile*, and *exp(·)* is the exponential function. We computed the MOR using a Stata command: nlcom exp(sqrt(2*_b[/var(_cons[kebele_BaselineM])])*invnormal(0.75)), cformat(%9.2f) and the result was greater than 1 which showed a considerable between-cluster variation to proceed with the multilevel analysis [24]. In addition, the Akaike information criteria (AIC) and loglikelihood model were used in comparison with other models to estimate models goodness-of-fit (Table 5).

Collinearity was assessed using the means of variance inflation factors (VIFs) as a post-estimation procedure, following for regression analysis because this study used several explanatory variables that might be correlated to each other (such as mother’s education, husband’s education and the household wealth index). The multi-collinearity between both parents’ education levels was found to be significant, so the husband’s education was excluded from the regression models (Variance Inflation Factor > 5).

## Results

### Respondents’ characteristics

A total of 727 pregnant women participated from rural areas, making the response rate 99.3%. The participants’ mean age was 28.3 (± 4.3) years, and most of the women (77.6%) were 25–34 years old. The Gamo ethnic group was the predominant one in the sample (76.7%), with 42% living in the highlands. Regarding education level, 63% did not have any formal education. Most of the participants (66.8%) were Protestant Christians. The household wealth index was distributed equally, with 20% in the first three quintiles and 19% in the last two quintiles. About 76% of the study participants mentioned difficulty in accessing transportation in case of emergencies, and 44% had to walk on foot more than two hours to access health services [Table 3].

**Table 3:** Proportion and bivariate analysis result of the use of maternal health care services in southern Ethiopia

			**Received any ANC**		**Skilled attendance at delivery**		**Received PNC within 2 weeks**		
		***N***	**%**	**COR (95% CI)**	**%**	**COR (95% CI)**	**%**		**COR (95% CI)**
All respondents		727	63.4		46.5		33.4		
Individual and Household level variables									
Mother’s age	30–49	238	55.5	1	39.1	1	29.4		1
	15–29	489	67.3	1.6(1.2–2.3)	50.1	1.5(1.1–2.1)	32.5		0.8(0.5–1.1)*
Mother’s level of education	No formal education	504	52.9	1	35.7	1	23.2		1
	Formal education	223	87.0	5.9(3.8–9)	70.8	4.4(3.1–6.1)	50.2		3.2(1.6–3.1)
Mother’s occupation	Unemployed /housewives	662	62.4	1	44.9	1	30.1		1
	Farmers/Laborer	65	73.8	1.7(0.9–3)*	63.1	2.1(1.2–3.5)	46.1		1.5(0.9–2.6)*
Mother’s Religion	Orthodox	178	52.8	1	33.1	1	23.0		1
	Protestant	549	66.8	1.8(1.3–2.5)	50.8	2.1(1.4–2.9)	34.2		0.7(0.5–1.1)*
Mother’s Ethnicity	Gamo( Ref)	558	58.8	1	39.4	1	32.4		1
	Zeyse	144	76.4	2.2(1.4–3.4)	65.3	2.2(1.5–3.4)	18.7		0.6(0.4–1.0)
	Other**	25	92	8(1.8–34)	96.0	8(1.8–34)	84		6.2(2.4–15)
Gravidity	Multigravida( Ref)	513	63.1	1	47.6	1	31.4		1
	Grand-multigravida	214	64.0	1(0.7–1.4)*	43.9	0.9(0.6–1.2)*	31.8		1(0.7–1.4)*
Pregnancy was wanted	No	90	56.6	1	48.9	1	34.4		1
	Yes	637	64.4	1.3(0.8–2.1)*	46.1	0.8(0.6–1.3)*	31.1		1(0.6–1.6)*
Attitude towards Skilled care	Poor( Ref)	178	35.9	1	20.8	1	12.9		1
	Good	549	72.3	4.6(3.2–6.6)	54.8	4.6(3.1–6.8)	37.5		1.9(1.3–2.8)
Awareness on danger signs	No	374	43.3	1	54.2	1	60.6		1
	Yes	353	84.7	7.2(5–10)	45.8	3.4(2.5–4.7)	39.4		3.1(2.3–4.3)
Awareness on BPCR	No	-	-	-	24.2	1	-		-
	Yes	-	-	-	72.2	8(5.8–11.3)	-		-
Practice BPCR	Less prepared	-	-	-	32.2	1	-		-
	Well prepared	-	-	-	72.9	5.7(4–7.9)	-		-
Household socio-economic status	Richest	144	89.6	1	82.6	1	56.9		1
	Rich	145	77.2	0.4(0.2–0.8)	57.2	0.3(0.2–0.5)	37.2		0.4(0.2–0.6)
	Medium	146	73.9	0.3(0.2–0.6)	45.9	0.2(0.1–0.3)	32.2		0.3(0.2–0.4)
	Poor	146	58.9	0.2(0.1–0.3)	40.4	0.1(0.1–0.2)	28.1		0.3(0.2–0.4)
	Poorest	146	17.8	0.02(0.01–0.05)	6.8	.02(0.01–0.03)	3.4		0.2(0.1–0.3)
Household decision	Husband/ /respondents	231	50.6	1	29.9	1	10.4		1
	Jointly	496	69.3	2.2(1.6–3)	54.2	2.7(1.9–3.9)	41.3		1.5(1.0–2.1)
Community level variable (Level-2)									
Climatic zone the women reside	Highland ( Ref)	310	40.3	1	16.8	1		6.8	1
	Midland	188	71.8	3.7(2.5–5.5)	60.11	7.4(4.9–11)		56.9	1.6(1.1–2.5)
	Lowland	229	87.8	10.6(6.7–16)	75.5	15(10–23)		44.1	2.9(2.0–4)
Travel time to the nearest BEmONC	> = 2 h( Ref)	325	54.8	1	36.9	1		33.2	1
	< 2 h	402	70.4	1.9(1.4–2.7)	54.2	2(1.5–2.7)		30.1	1(0.7–1.2)*
Easiness to get transport	Very difficult( Ref)	554	55.9	1	35.2	1		24.9	1
	Easy	173	87.3	5.4(3.3–8.7)	82.6	8.8(5.7–13)		52.6	3(2.1–4.3)

^*^p value > 0.05 non-significant variables

^**^ Other 25 = Wolayta 15, Konso 6 and Gofa 4

^***^ Basic Emergency Obstetric and Newborn Care

### Maternal health care service utilisation (ANC, PNC and SBA)

Altogether, 63.4% (CI:59.8- 66.8) of the women had at least one ANC visit during their previous pregnancies, of whom only 8% started visits during the first trimester and 32.4% had four or more ANC visits during their previous pregnancies. Three hundred and thirty-eight women (46.5%) (CI:42.8- 50) had given birth to their previous babies in health facilities, and the remaining were homebirths of which more than half were assisted by family members or traditional birth attendants (TBAs). One in every three women had at least one PNC visit (33.4% (CI:30–36.9)) following the most recent childbirth [Table 3].

**Fig. 2:**
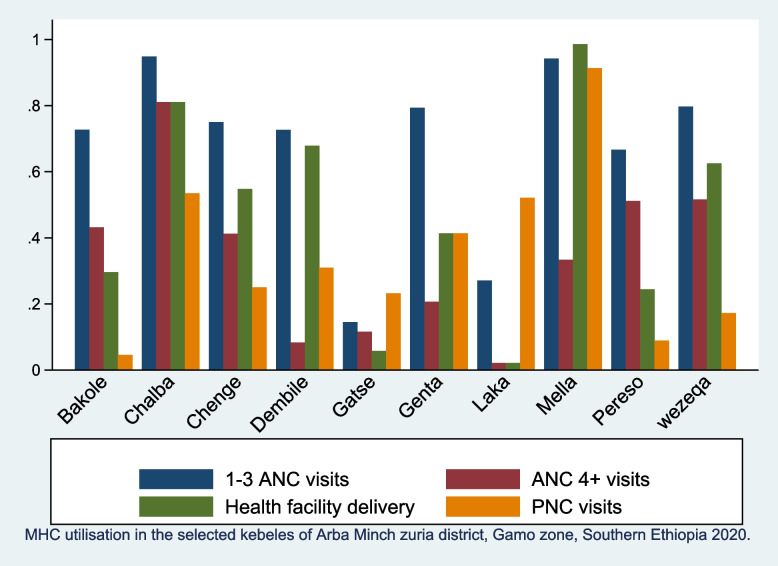
Proportion of Maternal health care utilization at the different clusters/kebeles in in Gamo zone, 2020

In terms of MHC service utilisation at the clusters (*kebele*) level, the Mella cluster (ANC 94%, SBA 98% and PNC 98%) and Chano cluster (ANC 94%, SBA 81% and PNC 48%) were found to have the highest MHC service utilisation, whereas the Laka cluster (ANC 27%, SBA 2% and PNC 2%) and Gatse cluster (ANC 14%, SBA 5.8% and PNC 1%) were found to have the lowest. Similarly, MHC service utilisation tends to be more common among lowland clusters with short distances from home to health facilities and less difficulties to access transportation than the counterpart clusters (Fig. 2)**.**

### Knowledge of danger signs and BPCR practices

Nearly one in two women knew at least three of the danger signs during pregnancy, while a slightly lower proportion (45.8%) had mentioned the three danger signs during delivery, and the lowest proportion (39.3%) were knowledgeable about danger signs during the postpartum period. Altogether, 46% of study participants were aware of BPCR, and 34% had saved money to cover costs during SBA or in the event of an emergency. One in every three respondents (36.7%) decided in advance where to give birth, and 34.2% of the women overall were prepared for birth and its possible complications (who met at least three criteria).

### Bivariate analysis results from maternal health care service utilisation

Bivariate analysis was performed for study variables that may have been predictors of MHC service use in the multilevel analysis. Factors that showed a positive association with ANC in a bivariate analysis were younger age (15 to 30 years old), having a formal education, Protestant religious affiliation, non-Gamo ethnic categories, a positive attitude towards skilled care, being knowledgeable about danger signs during pregnancy, being from the fifth household wealth quintile, being a lowlands resident, involved in decision-making to seek health care, having less difficulty to access transportation and needing to travel less than two hours to reach healthcare facilities [Table 3].

Younger age (15–30), Protestant religious affiliation, formal education attainment and employment, having a husband with a formal education and who is employed, being in the fifth wealth quantile, being involved in decision making, lowland residents, easy access to transportation, needing to travel less than two hours to reach healthcare facilities, ANC use, BPCR practice, being knowledgeable about danger signs during childbirth and a positive attitude towards institution-based skilled birth attendance all indicate a greater likelihood of health facility delivery.

Furthermore, an association also existed between PNC use and place of residence, maternal education, husband’s education and employment, household wealth quintile, women’s level of decision-making, ease of getting to a health facility, ANC use, being knowledgeable about danger signs during the postpartum period, positive attitude towards health facilities and SBA at health facilities.

### Multilevel analysis of determinants of maternal health care service utilisation

A multilevel binary logistic intercept-only model (Model 0) was used to test the null hypothesis that no variation exists in the use of MHC services between clusters (*kebeles*). Accordingly, for each indicator of MHC service utilisation, significant variation between clusters was detected. For ANC, SBA and PNC, the Intra Cluster Coefficient in the empty model revealed that differences between clusters/*kebele* accounted for 38%, 58% and 31% of the total variance in service utilisation, respectively. Tables 4 and 5 provide the detailed test of goodness of fit for the mixed-effects multilevel logistic regression for the three outcome variables and the predictors of MHC utilisation respectively.

**Table 4:** Parameter coefficients of the mixed-effects multilevel logistic regression for the three outcome variables—Empty model, without covariates

Empty model	ANC use	SBA use	PNC use
Random effects as community level variance	1.99	4.6	1.5
ICC	38%	58%	31%
MOR	2.5	3.9	2.2
AIC	733	719.8	804
Log likelihood (LR) deviance	364.8	357.9	400
Wald χ2	Reference	Reference	Reference
Significance of LR test vs. logistic regression (*p* value)	< 0.0001	< 0.0001	< 0.0001

The empty model contains no variables but partitions the variance into two component parts

**Table 5:** Multilevel logistic regression analysis of the determinants of MHC services utilization among pregnant women in southern Ethiopia,2022

		Received any ANC			Skilled attendance at delivery			Received PNC within 2 weeks		
All respondents		Model 1 (level-2 variables) AOR (95% CI)	Model 2 (level 1 variables) AOR (95% CI)	Model 3 (full model) AOR (95% CI)	Model 1 (level 2 variables) AOR (95% CI)	Model 2 (level 1 variables) AOR (95% CI)	Model 3 (full model) AOR (95% CI)	Model 1 (level 2 variables) AOR (95% CI)	Model 2 (level 1 variables) AOR (95% CI)	Model 3 (full model) AOR (95% CI)
Individual level variables										
Mother’s age	30–49	-	1	1	-	1	1	-	-	-
	15–29	-	1.4(0.8–2.2)	1.3(0.8–2.2)	-	1.1(0.6–1.7)	0.9(0.6–1.6)	-	-	-
Mother’s education	No formal education	-	1	1	-	1	1	-	1	1
	Formal education	-	1.5(0.8–2.8)	1.6(0.9–3.1)	-	1.4(0.8–2.4)	1.7(0.9–3.1)	-	0.9(0.5–1.4)	1.1(0.6–1.7)
Mother’s occupation	Unemployed	-	-	-	-	1	1	-	-	-
	Farmers/Laborer	-	-	-	-	3.5(1.5–7.9)*	3.1(1.3–7.3)*	-	-	-
Mother’s Religion	Orthodox	-	1	1	-	1	1	-	-	-
	Protestant	-	0.5(0.3–1.03)	0.6(0.3–0.9)	-	0.8(0.5–1.5)	0.7(0.4–1.3)	-	-	-
Mother’s Ethnicity	Gamo( Ref)	-	1	1	-	-	-	-	1	1
	Zeyse	-	3.8(0.7–17)	1.3(0.3–6.1)	-	-	-	-	0.7(0.1–3.4)	0.6(0.1–3.9)
	Other*	-	2.2(0.3–14)	0.9(0.1–6.3)	-	-	-	-	0.9(0.2–3.3)	0.8(0.2–3.3)
Pregnancy was wanted	No	-	1	1	-	-	-	-	-	-
	Yes	-	3.9(1.8–8)*	3.9(1.8–8.3)*	-	-	-	-	-	-
Attitude towards Skilled care	Poor( Ref)	-	1	1	-	1	1	-	1	1
	Good	-	0.9(0.-1.6)	0.8(0.5–1.6)	-	1.5(0.9–2.8)	1.6(0.9–2.9)	-	1.1(0.6–1.9)	1.1(0.6–1.9)
Awareness on danger signs	No	-	1	1	-	1	1	-	1	1
	Yes	-	6.8(3.8–12)*	6.8(3.8–12)*	-	1.5(0.8–2.6)	1.6(0.2–2.9)	-	0.9(0.4–1.7)	0.7(0.3–1.4)
Household economic status	Richest	-	1	1	-	1	1	-	1	1
	Rich	-	0.9(0.4–2.2)	1.1(0.5–2.6)	-	0.6(0.3–1.3)	0.5(0.2–1.2)	-	0.8(0.4–1.8)	0.8(0.4–1.8)
	Medium	-	1.1(0.4–2.7)	1.3(0.5–3.3)	-	0.6(0.2–1.4)	0.6–0.2–1.4)	-	1.0(0.4–2.3)	0.9(0.4–2.2)
	Poor	-	0.5(0.2–1.3)	0.6(0.2–1.7)	-	0.4(0.2–1.1)	0.5(0.2–1.4)	-	0.9(0.4–2.3)	0.9(0.3–2.3)
	Poorest	-	0.4(0.1–1.1)	0.4(0.1–1.3)	-	0.1(.05–0.6)*	0.3(0.1–1.0)	-	0.6(0.2–1.5)	0.6(0.2–1.7)
Household decision	Husband/women	-	1	1	-	1	1	-	1	1
	Jointly	-	1.0(0.6–1.7)	1.03(0.6–1.8)	-	1.3(07–2.5)	1.4(0.7–2.9)	-	0.7(0.4–1.2)	0.7(0.4–1.2)
Climatic zone	Highland ( Ref)	1	-	1	1	-	1	1	-	1
	Midland	0.8(0 .3–1.9)	-	1.9(0.6–5.7)	0.7(0.2- 0.7)	-	1.5(0.5–4.4)	4.4( 1.6- 11)*	-	4.7(1.7–13)*
	Lowland	1.9 ( 0.5- 6.5)	-	4.1(1.1–14)*	0.2(0.1–0.6)	-	0.3(0.1–1.5)	0.7(0.1- 2.7)	-	0.9(0.2–3.4)
Travel time nearest BEmONC	> 2 h( Ref)	1	-	1	1	-	1	-	-	-
	< 2 h	1.8(1.1–3.2)*	-	1.5(0.5–3.8)	3.1( 1.5- 6)	-	2.9(1.4–5.8)*	-	-	-
Easiness to get transport	Very difficult( Ref)	1	-	1	1	-	1	1	-	1
	Easy	2.6(1.0,6.5)*	-	1.9(1.1–3.7)*	14(5.4–39)	-	10(3.6–29)*	2(1.1–4.4)*	-	1.5(0.7–3.3)
BPCR practice	Less prepared	-	-	-	-	1	1	-	1	1
	Well prepared	-	-	-	-	0.8(0.5–1.6)	0.7(0.4–1.4)	-	1.2(0.6–2.3)	1.1(0.5–2.1)
ANC use	No	-	-	-	-	1	1	-	1	1
	Yes	-	-	-	-	3(1.7–5.3)*	3.3(1.8–5.9)*	-	0.7(0.5–1.3)	0.9(0.5–1.6)
SBA	No	-	-	-	-	-		-	1	1
	Yes	-	-	-	-	-		-	2.4(1.5–3.9)	2.1(1.2–3.5)*
Test of goodness-of-fit of the multilevel logistic regression model	Random effects as *kebele level* variance	1.3	1.25	0.7	5.9	2.8	2.6	2.1	1.3	1.8
	MOR	2.1	2.1	1.7	4.7	2.9	2.8	2.4	2.1	2.4
	AIC	725.8	647.6	644.4	668.3	675	636	784	809	796
	VPC = ICC (%)	28%	27%	18%(	64%	46%	45%	36%	27%	35%
	Log likelihood (LR) deviance	356	308.8	303.2	328	322	299.2	387	389	380
	Wald χ2	15	84.7	95.0	46.4	61.2	86.0	22	19.8	34.5
	LR test vs. LR (p value)	< 0.0001	< 0.001	0.0003	< 0.001	< 0.001	< 0.001	< 0.001	< 0.001	< 0.001

Dependent variable: ANC/SBA/PNC; (ii) cluster variable: kebele (9 in number); Model 1: cluster level variables included; Model 2: individual level variables included; Model 3: full model (all the cluster level and individual level variables included; * *p* < 0.05 shows statistically significance; VPC = Variance Partition Coefficient; AIC = Akaike Information Criterion

### Antenatal care (ANC)

Being knowledgeable about danger signs during pregnancy and pregnancy planning were strong predictors of ANC use at the individual level. Women who are knowledgeable about the danger signs during pregnancy were 6.8 times more likely to use ANC compared with non-knowledgeable women. Similarly, women who reported that their previous pregnancies were planned were about four times more likely to have ANC than women whose pregnancies were mistimed or unplanned. The study also found that community-level factors (*kebeles*), such as easy access to transportation and residing in lowlands areas, were important predictors of ANC utilisation. At the community level, residing in lowlands areas was associated significantly with ANC use, with lowlanders 4.1 times more likely to use ANC than highlanders. The ease of getting access to transportation in a community cluster also was found to be a key factor in ANC utilisation. The likelihood of utilising ANC was about two times higher in clusters with easy access than in clusters without easy access.

### Skilled birth attendance (SBA)

Concerning SBA, maternal employment status and ANC use were found to be significant individual-level predictors. Compared with housewives, employed women were 3.1 times more likely to use SBA. Similarly, ANC use was a strong predictor of SBA, i.e., women who received ANC were 3.3 times more likely to give birth in a health care facility than those who had not received ANC.

The two second-level predictors – the presence of a health centre within two hours of travel distance and transportation access to the health facility – were associated significantly with SBA use. Women who lived in communities with health centres (BEmONC) within two hours in travel time were 2.9 times more likely to use SBA than women living farther away. The ease of getting access to transportation in a community cluster also was found to be a key factor in SBA utilisation. The likelihood of giving birth at healthcare facilities was 10 times higher in clusters with easy access to health care facilities than in clusters without easy access.

### Postnatal care use (PNC)

In this study, two predictor variables were found to be significantly associated with the use of PNC – one at the individual level and one at the cluster level. At the individual level, when compared with women who gave birth outside the health institutions, women who had given birth at healthcare facilities were 2.1 times more likely to receive PNC services. At the cluster level, being from the midland area indicated a significant increase in PNC utilisation, with midlands residents 4.7 times more likely to receive PNC services than highlanders.

## Discussion

This study examined the relationship between individual and community-level factors concerning MHC service uptake (ANC, SBA and PNC). The results revealed that the uptake of the three relevant MHC services was low in this study area compared with the national target set for 2020 [25], and with other African nations’ MHC service indicators [8]. An estimated 63% of the pregnant women in the present study visited ANC services, which was lower than the corresponding result of nearly 70% in rural areas reported in EDHS 2019 [9]. However, the finding is consistent with a pooled point prevalence of ANC utilisation in Ethiopia, which was 63.7% [26].

The proportion of women who had given birth at healthcare facilities was estimated at 46.5% in the present study, but all the births that occurred outside of healthcare facilities were assisted by family members/TBAs, who may not have the skills and resources to manage life-threatening complications that can arise during labour and the immediate postpartum period [27]. Furthermore, the high number of home births could be a contributor to poor maternal health in the district. Therefore, relevant initiatives should be implemented to promote the use of SBA to help reach the national target of 90% SBA nationwide [25]. About 33.4% received PNC services, which is slightly higher than the 28.8% that EDHS [9] reported for rural women, as well as percentages from other studies conducted in Ethiopia [28] and Zambia [29]. However, this finding is consistent with a point prevalence from a systematic review in Ethiopia (32%) [30] and in a study from Nigeria [31]. The difference could be due to the fact that we included all visits to the health facility after childbirth, including health check-ups and immunisations, under the assumption that every visit to the health care facility will encourage women to seek care at the time of contact. However, overall use remains very low, which is likely due to the high number of home births in this rural district [32].

At the individual level, awareness about the pregnancy danger signs was found to be positive predictor to receive ANC services. This finding is consistent with previous studies that found knowledge of danger signs during pregnancy had a statistically significant association with the utilisation of MHC services [30, 33, 34], possibly because increased awareness of potential risks during pregnancy and delivery may induce behavioural changes, such as health-seeking behaviour, which includes seeking professional care [8]. Thus, working to raise awareness of danger signs during pregnancy and postpartum, as well as educating women about MHC services’ importance, would enable women to take advantage of existing services in this rural district [35].

Moreover, the likelihood of ANC visits also was significantly higher for women with planned pregnancies, which is consistent with findings from studies conducted in other parts of Ethiopia [11, 33, 34, 36]. One possible explanation could be that women with unplanned or mistimed pregnancies devote less attention to the pregnancy and the care required for it [26, 37]. It also is agreed that unplanned or mistimed pregnancy as a determinant in infrequent use of ANC has received less attention than other individually related barriers [38, 39]. This study suggested that efforts to promote family planning among rural women to minimise unintended pregnancy not only may increase MHC service uptake, but also may reduce unsafe abortions, which is one of the country’s leading causes of maternal mortality [38, 40].

The study had also showed that women who engaged in farming/labourer were more likely to receive SBA during delivery than housewives’. This finding was consistent with other studies conducted in Ethiopia [18, 32] that also found employment is likely to enhance women’s status, helping them develop greater confidence to make decisions about their health, such as accessing healthcare facilities. Moreover, employed individuals were more likely to overcome financial constraints, which are a typical obstacle to obtaining MHC services in rural Ethiopia. Also, working women have easier access to information, which helps close knowledge gaps and creates positive attitudes that encourage women to seek MHC services [11]. Thus, creating local opportunities from which women could benefit financially is important to improving access to existing services in the area [41].

ANC was a significant predictor of receiving SBA during delivery [11, 13, 14]. ANC offers pregnant women the opportunity to learn about preparing for birth and the benefits of childbirth in a healthcare facility, which, in turn, can influence their decision to use SBA [34]. While we could not find any significant difference in BPCR practice concerning SBA use, having a personalised BPCR plan that will help women prepare for potential emergencies during the childbirth process is advisable. However, not all women who used ANC also used SBA. The women may be reluctant to seek assistance from health care institutions if they believe their pregnancies are normal, or may experience access difficulties, including labour that begins late at night, when the women couldn’t get transportation to healthcare facilities [11, 13, 14]. Mothers who had given birth at a healthcare facility and had used ANC services were more likely to get counselling about PNC and danger signs after childbirth, which may encourage them to consider using existing services [28, 30, 42–44].

Women living in the lowlands were more likely to use ANC services compared with those in the highlands, who were more likely to have poor access to health services, poor infrastructure and longer distances to travel for health care. Moreover, a sociocultural difference such as traditional belief systems, access to education and wealth status also might hinder ANC use among highlanders [45]. Similarly, women living in Midland areas were more likely to use PNC than highlanders. The poor socioeconomic status of women in the highlands, as well as the presence of more health services in the lowlands, could explain this result. However, this finding also points to the need for a contextual and localised intervention that will benefit all women in the area [12, 46].

According to a systematic review from developing countries, distance and increased travel time to the nearest health care facilities have been found to be associated significantly with ANC and SBA use [46]. Similarly, ANC and SBA uptake was lower in communities where the healthcare facility was difficult to reach [21, 46, 47]. Consistently, this study found that having easy access to a health facility made a considerable impact on ANC service use, as limited access to health facilities negatively affects health-seeking behaviour and may have resulted in low ANC use. Similar to most developing countries – in which most infrastructure is concentrated in urban areas and is scarce in rural areas, where most of the population lives – the disparity makes it difficult for women, particularly those living in rural areas, to access health care. While efforts aimed at closing the gap between rural and urban areas are encouraged, working on improving individual women’s access is suggested [21, 47].

The study also indicated that women who lived in communities with health centres (BEmONC) within two hours in travel distance and who had easy access to transport were more likely to use SBA. Other studies also found similar results elsewhere in the same context [47, 48] and in developed countries [49]. Women who must travel for more than two hours and who had difficulty accessing transport were less likely to give birth in a health centre. This result suggests that a physical accessibility problem exists for rural women who want to use existing healthcare facilities. Many women in rural areas must walk long distances on difficult terrain or be carried on a traditional stretcher to reach a health centre. This journey through the hills under difficult geographical and health circumstances leaves many women either opting to give birth at home or giving birth on the side of the road when they cannot reach a facility in time, likely increasing the risk of complications or death for both the mother and the unborn baby [50].

Making these services physically accessible through the establishment of maternity waiting homes (MWHs), which are facilities that house pregnant women during the final few days or weeks of their pregnancies, provides easier access to nearby healthcare facilities and is one of the common practices in developing countries, including Ethiopia [51, 52]. However, even though MWHs are one of the mitigation strategies for addressing disparities, most MWHs suffer from a lack of quality care and were not integrated into the health care system in Ethiopia [52, 53]. As a result, integrating MWHs into the health system while maintaining an explicit link to the community should make it easier for women living in rural and remote areas to access facilities [54].

### Strengths and limitations

This study’s sample was drawn from intervention and comparison areas for the evaluation of a community-based intervention to improve the use of MHC, which was implemented after a complete census of pregnant women in Gamo Zone’s Arba Minch Zuria district in Southern Ethiopia. Previous research has focussed on one or a few aspects of MHC services, but we evaluated the three relevant MHC indicators and tried to identify determinants at individual and community levels to provide a complete picture of maternal health, as well as valuable information for policymakers that can be used when planning context-specific interventions.

However, the findings should be interpreted in light of several limitations. Considering that the data were self-reported, particularly distance and travel time, this was prone to recall and social desirability bias, which could have influenced the data’s internal validity. Recall bias was reduced by focussing on the most recent birth during the past five years, and the interviews were conducted in private places to reduce social desirability bias and reassure participants on data confidentiality. Moreover, causal inferences are not possible with observational data examined in this study. Furthermore, the proxy used for distance to health facilities did not measure actual distance, but rather derived it from respondents and data collectors’ estimates. The extent to which this proxy variable truly reflects the distance to services is uncertain.

## Conclusions

MHC service utilisation in the study area can be viewed as low, with approximately two-thirds of pregnant women receiving ANC, half utilising SBA and one-third utilising PNC. At the cluster (*kebele*) level, being a lowlands resident, having easy access to transportation and living near a health facility were all linked to a higher likelihood of using MHC services. Moreover, we found a significant cluster-level variation in the utilisation of the three MHC indicators in the area.

Knowledge about danger signs during pregnancy also was found to be a predictor of ANC use among individual-level factors. Thus, designing context-specific interventions to address distance and improve access to MHC services, with a particular emphasis on enhancing and strengthening the maternity waiting homes programme, needs to be prioritised. Improving women’s participation in socioeconomic activities would yield greater results in increasing the use of MHCs. Strengthening promotion of maternal health care through community health education, boosting community awareness programmes with an emphasis on obstetric danger signs of pregnancy and family planning will be important in improving MHC service utilisation. Moreover, it is very important to promote ANC utilisation, which increases the odds of SBA and PNC use and encourages women to have individualised birth plans. To increase the utilisation of MHC services in the district, in addition to interventions targeting individual-level factors, a strong need exists to focus on community and district-level interventions, with a particular emphasis on the highland clusters. Future research also is needed to examine factors that can explain cluster-level variations in MHC service use and examine in depth the reasons for huge differences among clusters.

## Supplementary Information

**Additional file 1. **

## Data Availability

All data generated during this study are included in this published article and its supplementary information files. The dataset for this study are available from the corresponding author upon reasonable request.

## Abbreviations

ANC: Antenatal care
BPCR: Birth Preparedness and Complication Readiness
LMIC: Low and Middle Income Countries
MHC: Maternal Health care
ODK: Open Data Kit
PNC: Postnatal Care
SBA: Skilled Birth Attendance
WHO: World Health Organization
